# Recreating cellular dedifferentiation processes using weak electric fields

**DOI:** 10.3389/fbioe.2026.1927666

**Published:** 2026-10-28

**Authors:** P. Van Blerk, P. Potgieter, L. Linde, C. F. Birkill

**Affiliations:** Algiamed Technologies, Burnaby, Vancouver, BC, Canada

**Keywords:** cell differentiation, dedifferentiation, *in-vitro* experiments, pluripotent stem cells, red blood cells, replication study

## Abstract

Bioelectrical signals play a crucial role in controlling the behaviour and physiological processes of various types of cells, including the differentiation of stem cells into more specialised cell types. The potential for reversing the differentiation process through the application of bioelectrical signals could potentially allow the induction of pluripotent stem cells from differentiated cells. In hopes of expanding the existing body of research, the primary objective of this study was to replicate the *in-vitro* experiment conducted by Becker and Murray in 1967. The experiment focused on investigating the induction of cellular dedifferentiation in nucleated amphibian red blood cells through the application of small electrical currents. The results obtained substantiated Becker and Murray’s findings and offer further insights into the factors that potentially contribute to the process of cell dedifferentiation. This study highlights the need for further research to refine the mechanisms and technical aspects of electrically inducing dedifferentiation as well as explore the possibility of using small electric fields as a means to guide the redifferentiation process.

## 1 Introduction

Endogenous bioelectrical field gradients are a fundamental regulator of cellular behavior, governing processes ranging from proliferation and directed migration (electrotaxis) to large-scale morphogenesis and regeneration (Levin, 2014; McCaig et al., 2005; Song et al., 2007). These fields arise largely from the unequal distribution of ions across cell membranes, acting as instructive signals that initiate downstream biochemical cascades and gene expression (Nuccitelli, 2003; Funk, 2015). In regenerative species such as amphibians, this bioelectric control is functionally critical. For instance, the well-characterised ‘skin battery’ effect generates an outward current through the stump tissue of an amputated limb. This bioelectric gradient is not merely a byproduct of injury but an active guidance cue; it is required to direct the galvanotaxis of macrophages and neutrophils to the wound site, orchestrating the release of cytokines and growth factors necessary for tissue repair (Hackam and Ford, 2002; Altizer et al., 2002; Borgens et al., 1984). Consequently, there is significant interest in applying exogenous currents to mimic these cues to manipulate cell fate, a concept supported by recent success in electrically promoting tissue regeneration in mammalian models (Leppik et al., 2015). While mammalian fracture healing relies predominantly on the proliferation of resident periosteal stem cells, amphibian regeneration utilizes a distinct and more plastic mechanism: the de-differentiation of mature, nucleated erythrocytes into less-specialised precursor-type cells with fibroblastic potential. Becker and Murray (1967) identified that this process is governed by the specific bioelectric environment of the fracture hematoma. Their phylogenetic investigation revealed that a rapid, injury-proportional shift toward negative periosteal potential within the first 2 hours of injury serves as the trigger for this cellular transformation. They demonstrated that this *in vivo* phenomenon could be replicated *in vitro* by exposing erythrocytes to low-magnitude currents. They reported that very small electrical currents applied through a non-uniform direct-current field induced morphological and biochemical changes associated with dedifferentiation in mature, non-dividing nucleated erythrocytes, without the use of genetic manipulation or conventional biochemical reprogramming factors. This finding preceded the development of genetically induced pluripotency by approximately 4 decades and, if independently reproducible, could provide a distinct experimental model for investigating how bioelectrical signals influence the state of differentiated cells. Their findings indicated a ‘window of efficacy’ for dedifferentiation, where currents between 200 and 300 pA successfully triggered cytoskeletal reorganization and nuclear activation. This response exhibited a distinct spatial progression influenced by electrode polarity, while currents outside this range failed to induce the change or caused cellular damage. This implies that cellular plasticity can be modulated solely through bioelectric parameters, offering a physiological alternative to the more complex interventions required in induced pluripotent stem cell (iPSC) generation (Takahashi and Yamanaka, 2006). Despite the foundational nature of the Becker and Murray (1967) findings and their potential implications for regenerative biology, the limited independent investigation of this phenomenon leaves its reproducibility in need of further verification. Several subsequent studies have examined related electrically or electromagnetically induced responses in nucleated erythrocytes. Harrington and Becker (1973) reported that electrical stimulation of frog erythrocytes was accompanied by RNA and protein synthesis, while Smith et al. (1978) presented evidence suggesting that transmembrane calcium movement contributed to the response. Chiabrera et al. (1979) used cytofluorometric methods to investigate electromagnetically induced changes in frog erythrocytes, and Imani et al. (2019) reported frequency-dependent morphological changes in nucleated avian erythrocytes exposed to extremely low-frequency electric fields. However, these studies differed in species, stimulation waveform, field configuration and analytical methods, and none appears to constitute a close independent replication of Becker and Murray’s original low-current, non-uniform direct-current protocol. Consequently, no routinely used or standardised method for inducing cellular dedifferentiation using very small electrical currents has been established. The primary objective of this study is to revisit the experimental framework established by Becker and Murray (1967) in a preliminary, proof-of-concept investigation. Specifically, this research explores the morphological response of amphibian erythrocytes to low-magnitude currents within a non-uniform field. By isolating this bioelectric variable in a controlled *in vitro* setting, this study aims to assess the potential for replicating non-genetic cellular reprogramming and provide preliminary data to inform future, high-fidelity investigations into electrically mediated regeneration.

## 2 Brief summary of Becker and Murray’s (1967) article

In 1967 Becker and Murray used nonuniform field chambers and electrical stimulation within certain current limits to successfully replicate the sequence of morphological changes observed at amphibian fracture sites. The observed dedifferentiation phenomenon seemed to be contingent on several variables, including the total current level, degree of field non-uniformity, and the duration of exposure. A relationship with the total current level could be established by equating the efficiency of the electrical factors to the time required to produce a complete sequence of morphological changes in a zone within 1 mm of each electrode. From these results, it was observed that the most favourable current level for the chambers ranged between 200 and 300 pA. The efficiency of the chambers diminished when the current level exceeded or fell below this range. In each instance, there was a systematic spatial progression of cellular alterations, initiating in the areas of highest field non-uniformity. Over time, the changes spread further outward into the regions farther away from the electrode. The rate of morphological change in close proximity to the positive electrode was higher compared to that observed near the negative electrode (Becker and Murray, 1967).

## 3 Objective of this research

Substantiating Becker and Murray’s findings would prove that cell state can be modulated by bioelectrical signals alone, supporting the idea that bioelectric fields act upstream of gene expression. A replication study is particularly important because Becker’s claims are extraordinary, and lie at the intersection of regeneration, stem cell biology, and bioelectricity.

The present study was designed as a replication with refinement, aimed at verifying the robustness of Becker and Murray’s observations under more controlled conditions. Its primary goal was to examine the replicability of inducing cellular dedifferentiation in amphibian RBCs through the application of very small electrical currents *in-vitro* (Becker and Murray, 1967; Becker and Selden, 1998). By confirming these findings, this research serves as a validity check, clarifies critical experimental parameters, and provides a foundation for further investigation.

This replication was not intended as a comprehensive, fully controlled study designed to generate extensive datasets or systematically explore all possible parameters, but rather as a proof-of-concept investigation with a limited number of additional variables examined.

## 4 Materials and methods

### 4.1 Cell source and culture conditions

For Becker and Murray’s experiment fresh heart blood was collected from adult frogs that were sacrificed for the purpose of the experiment. This blood was diluted using balanced amphibian Ringer’s solution. Subsequently, the diluted blood was subjected to various electrical stimuli within small enclosed chambers, which were positioned directly on the stage of an inverted biological microscope, allowing continuous visual observation of the cells.

For this study amphibian red blood cells were collected from five raucous toads (*Sclerophrys capensis*) as well as three guttural toads (*Sclerophrys gutturalis*). Blood was collected from the ventral abdominal vein using a 0.3 mL syringe. A maximum of approximately 0.15 mL was collected from each animal, corresponding to no more than 10% of the estimated total blood volume of the frog. In some instances, the blood samples were sufficiently large for several tests. Depending on the experimental condition, samples were stored in EDTA-treated, lithium-heparin-treated or untreated blood-collection tubes. Samples intended for immediate use were subjected to electrical stimulation within 10 min of collection. Where testing was delayed, samples were stored in sealed blood-collection tubes at approximately 2 °C–8 °C for a maximum of 24 h. Before stimulation, the blood was diluted with balanced amphibian Ringer’s solution and prepared as a sealed wet mount. The erythrocytes were not cultured or expanded; they were maintained *ex vivo* for observation and electrical stimulation.

A wet mount technique was used to prevent the dehydration of the specimen. The slides were prepared by placing one drop of blood solution onto a glass slide, after which a cover glass sealed with petroleum jelly was placed over the diluted blood sample on the slide, sealing the edges so the cells could be observed for longer periods.

The slides were fastened to the microscope’s stage directly beneath the objective, and the microscope was focused on the blood cells for the duration of the experiment. A combination of aluminium and silver foil (contacts) was used to create surface electrodes on the glass slide. These electrodes were connected in series with the custom variable current power supply to apply an electrical current through the blood sample on the slide.

Increasing increments of direct current were applied via the electrodes until changes to the cell morphology suggestive of cell dedifferentiation were observed in the cells located in close proximity to the electrodes. During each experiment, approximately 30 erythrocytes were visible within a typical microscopic field at any one time and were observed continuously during electrical stimulation, although each sample contained several hundred cells in total. Morphological assessment was qualitative and based on direct visual observation. Cell and nuclear dimensions and nucleus-to-cell or cytoplasm-to-cell area ratios were not quantitatively measured.

### 4.2 Electrical current stimulation setup

Becker and Murray used a 1.5 V dry-cell battery, 3 mega ohm variable resistor, 10 mega ohm fixed resistor, and a Hewlett-Packard 425A microammeter that was placed in series with the test chamber which contained red blood cells diluted with balanced amphibian Ringer’s solution.

For this study, a custom precision adjustable current source was designed to provide electrical stimulation, with an output capability ranging from 50 pA to 300 nA. The circuit consisted of a Lithium Manganese Dioxide 9V battery coupled with an op-amp circuit and a variable resistor to adjust the current (Figure 1). A photograph of the experimental circuit setup is provided in Supplementary Figure S1.

**FIGURE 1:**
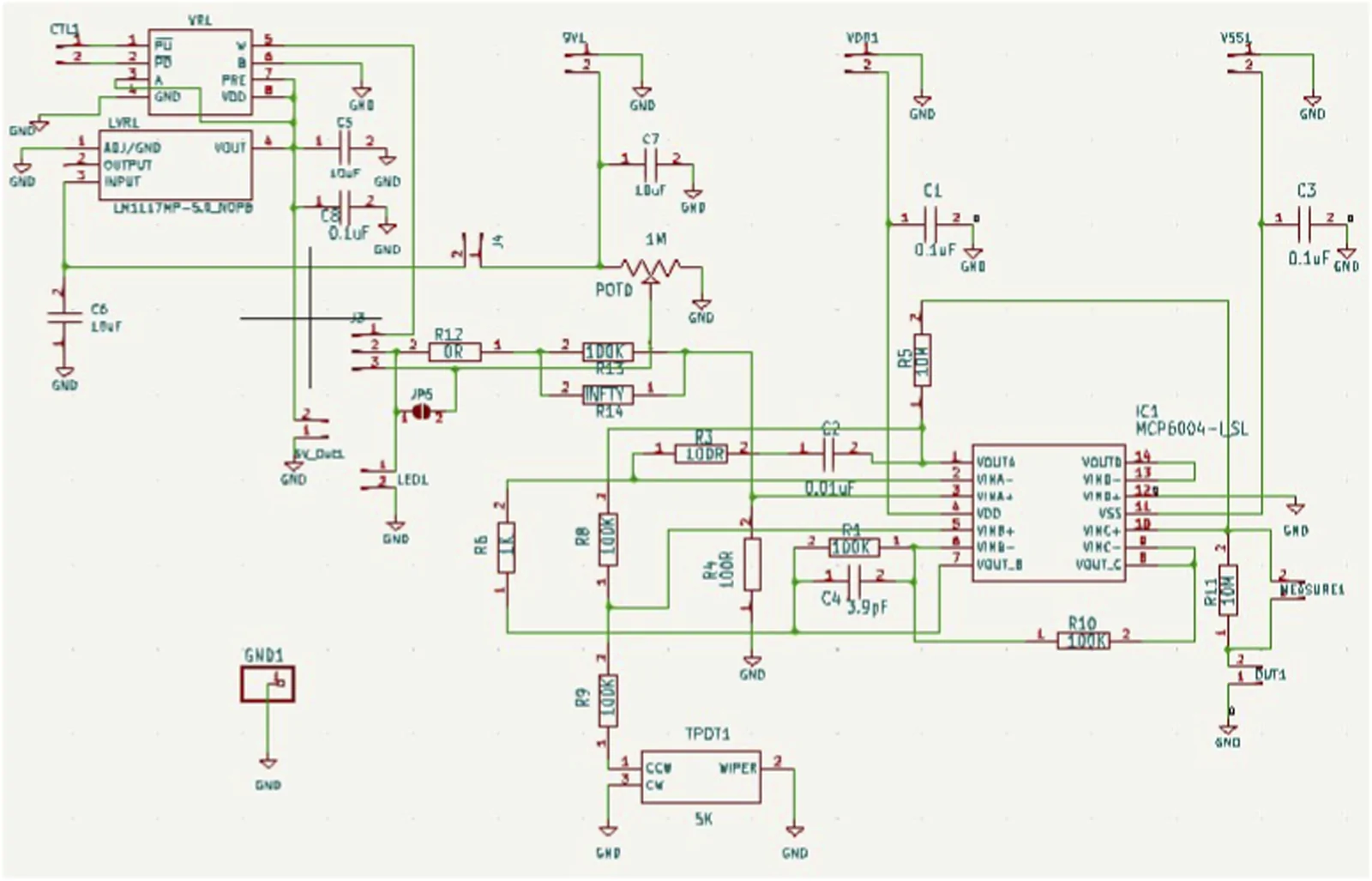
Custom-built precision adjustable current circuit used to deliver small direct currents to amphibian erythrocytes during the experiments.

The output current was calibrated using two independent methods. First, the current was calculated by measuring the voltage drop across a precision reference resistor of known resistance and applying Ohm’s law. Second, the output current was measured directly using a calibrated reference ammeter connected in series with the load. The two methods yielded consistent measurements, supporting the accuracy of the reported output-current values.

### 4.3 Anti-coagulants and administration

For *in vitro* blood experiments, the blood is typically collected in specialised tubes or syringes that are treated with suitable anticoagulants to prevent blood clotting. Blood was collected in either ethylenediaminetetraacetic acid (EDTA) or lithium heparin-treated tubes for this study. EDTA is frequently recommended as the preferred anticoagulant for haematological testing due to its ability to effectively preserve the integrity of cellular components and the morphology of blood cells. It chelates divalent cations such as calcium, which are necessary for the coagulation process. By removing these cations, EDTA prevents the formation of blood clots and stabilizes the blood sample for subsequent analysis (Schuff-Werner et al., 2013).

Lithium heparin is an anticoagulant that activates antithrombins, blocking the coagulation cascade of the blood sample, and is often recommended as an anticoagulant for various analyses involving whole blood or plasma samples due to its limited chelating properties, negligible impact on water shifts, and relatively low concentration of cations. It exhibits a lower probability of interfering in the outcomes of tests conducted for other ions, such as sodium (Wei et al., 2010).

Both types of anticoagulants, as well as samples using no anticoagulants, were employed in this study to ascertain the impact of the anticoagulant type on the response and morphological alterations of blood samples when subjected to small electric fields.

## 5 Improvements and limitations

A few key methodological improvements addressed ambiguities in the original work. First, electrical stimulation was delivered using a precision current source, replacing Becker and Murray’s variable resistor circuit, ensuring reproducible and accurately measured stimulus parameters. Second, the role of anticoagulants was examined by comparing EDTA, heparin, and no anticoagulant, controlling for potential confounding effects of the addition of different ionic concentrations on erythrocyte morphology. These refinements enhance experimental reproducibility, validate critical aspects of the original findings, and provide a framework for further exploration of bioelectric-induced cellular reprogramming. A limitation of this proof-of-concept study was that quantitative assessment of cell and nuclear areas and their ratios relied on approximate measurements derived from the available microscopy images. Measurement accuracy was limited by image resolution, uncertainty in boundary identification and the geometric assumptions used to estimate areas. These measurements therefore provide preliminary morphometric evidence. Future studies should use higher-resolution imaging and standardised image analysis to measure cell and nuclear dimensions and area ratios before and after electrical stimulation, compare findings across independent biological replicates, and incorporate complementary biochemical and functional analyses to determine whether the observed morphological changes represent cellular dedifferentiation.

## 6 Results

The study demonstrated that applying small electrical currents in non-uniform field chambers induced morphological changes consistent with a dedifferentiation-like transformation in amphibian nucleated erythrocytes. Although the full output range of the custom stimulation circuit (50 pA–300 nA) was evaluated, morphological responses were observed only within the 15–300 nA range. The transformed cells exhibited a more spherical shape, reduced cytoplasm-to-cell ratio, a prominent nucleus and loss of cytoplasmic colour. Measurements derived from the available microscopy images indicated an estimated 39%–45% reduction in projected cell area and an 11%–30% increase in nuclear area. The nucleus-to-cell area ratio increased by approximately 7–14 percentage points, with a corresponding decrease in the cytoplasm-to-cell area ratio. The sequence of changes observed in the present study, together with the corresponding sequence reported by Becker and Murray, is shown in Figure 2. The numbered stages indicate approximate morphological correspondence and do not represent precisely matched time points. Stage 7 in Figure 2b may represent a slightly earlier point in the transformation than stage 7 in Figure 2a, as indicated by the greater residual cytoplasmic colour. Spatially, these transformations originated at the electrodes and progressed outward, with the onset and completion of changes occurring faster at the anode than the cathode. Temporally, the rate of change was faster near the anode compared to the cathode, with initial changes at the former preceding those at the latter by five to 10 minutes (at 200 pA). The electrical stimulus acted as a ‘trigger’; once a cell began the morphological transformation (indicated by refractile of phase-shifted nuclei), it completed the full observed sequence of morphological changes suggesting dedifferentiation, even after the current was turned off. The study also noted that individual cells and samples collected from different individual frogs displayed varying response times and sensitivities. This variability could not be attributed to a particular species. Although all subjects were identified as mature adults, their precise ages were unknown; therefore, the differences in cellular responses could not be correlated with age. The rate of the dedifferentiation process was significantly altered by the type of anticoagulant used. When EDTA was utilised, morphological changes initiated within just 5 min of electrical stimulation, with the majority of cells exhibiting less differentiated characteristics within 2 h. In contrast, when samples contained lithium heparin or no anticoagulant, the reaction was heavily delayed: changes only began after 2 h of stimulation and extended just 3 mm from the electrodes after 12 h (Figure 3).

**FIGURE 2:**
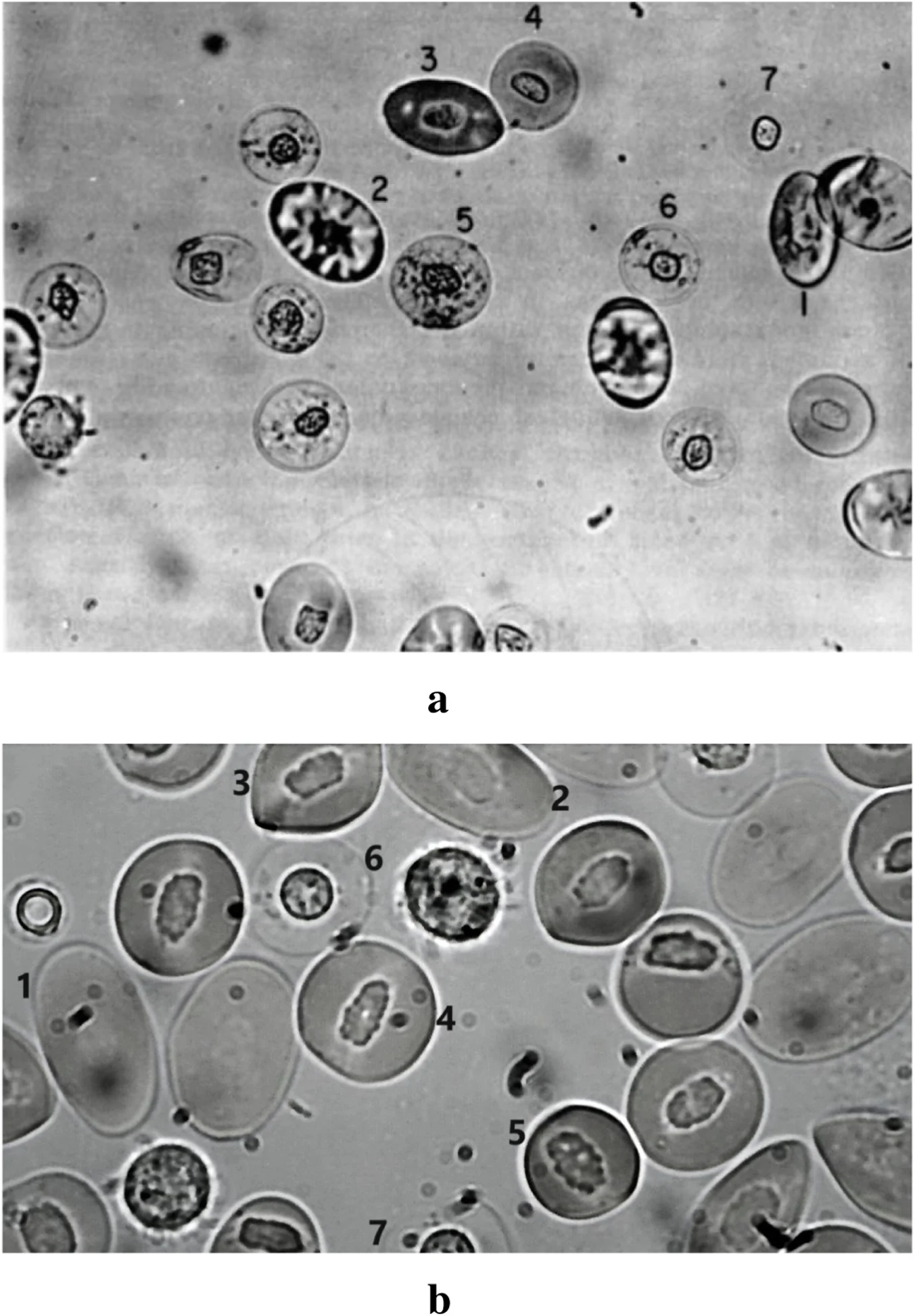
A representation of the typical morphological cellular changes produced by a small electrical field during **(a)** Becker and Murray’s experiments and **(b)** this study. The accompanying numbering denotes the order of morphological alterations, albeit with some missing stages. Notably, the observed sequence of cellular transformations aligns closely with the report on the maturation process from erythroblast to erythrocyte in amphibians by Holtfreter (1947).

**FIGURE 3:**
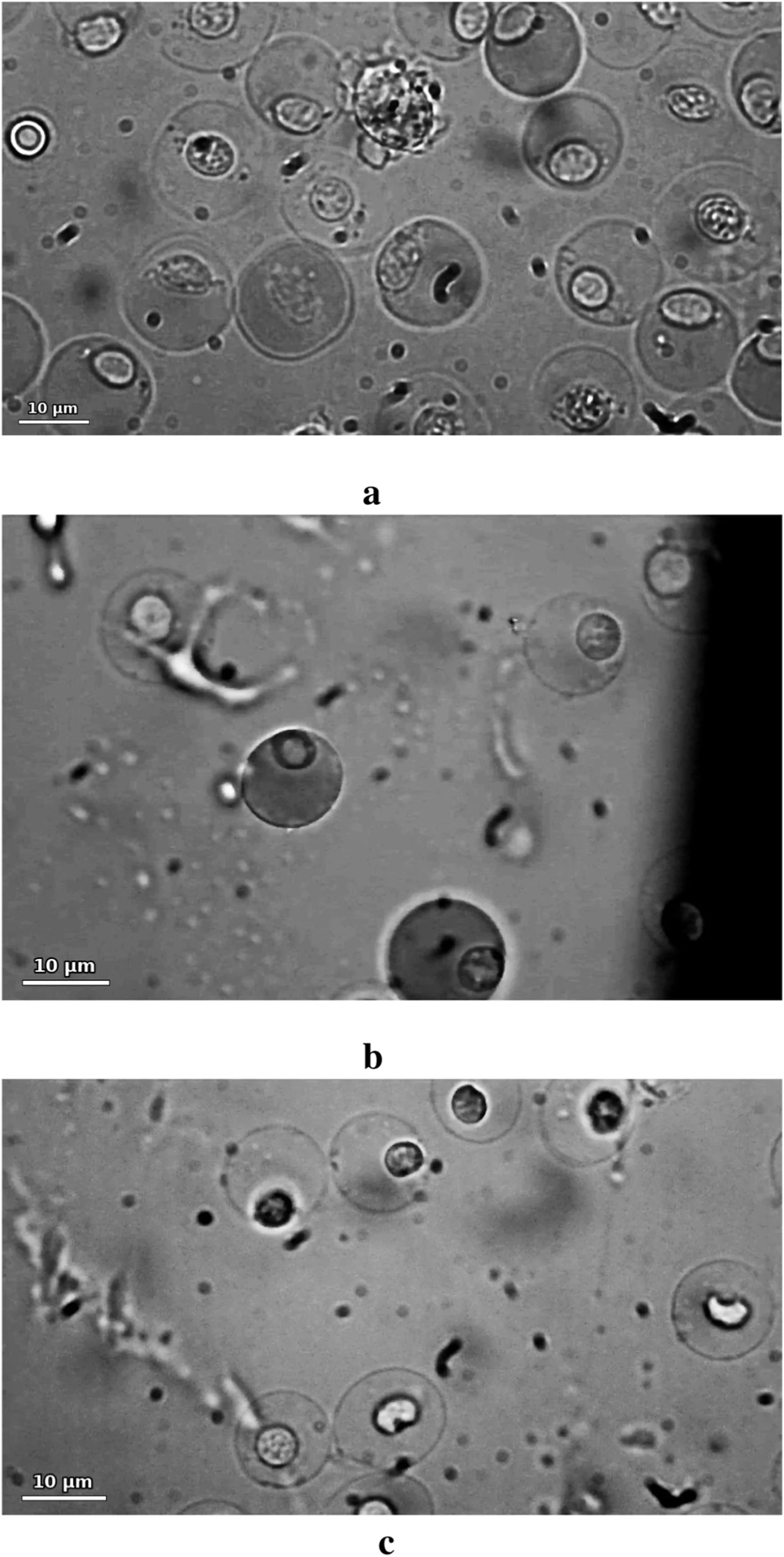
A representation of the typical morphological cellular changes produced in samples treated with **(a)** EDTA **(b)** lithium heparin and **(c)** no anti-coagulant.

## 7 Discussion

Both this study and the 1967 study by Becker and Murray indicate that the process of erythrocyte dedifferentiation in amphibians, a critical mechanism for regenerative healing, is influenced by electrical phenomena. These findings suggest that inducing pluripotent stem cells (iPSCs) with an exogenous electrical current and/or injected ionic “trigger-fluids” could offer a promising field of study. While limited by available funding and equipment, the study provides a foundational framework for future work, demonstrating that this phenomenon is reproducible and worthy of further investigation. It highlights the importance of exploring the mechanisms underlying bioelectric-induced dedifferentiation, the factors influencing the process, and potential implications for regenerative biology and clinical application. In both the present study and the predicate study by Becker and Murray, non-uniform field chambers were used to successfully create the morphological changes that are observed at amphibian fracture sites, specifically, suggesting that nucleated erythrocytes underwent a transition towards a less differentiated cellular state.

Several experimental and biological factors must also be considered when interpreting these findings. Cells exhibiting morphological changes became more spherical and showed a reduced cytoplasm-to-cell area ratio, increased nuclear prominence and progressive loss of cytoplasmic colour. These observed features are consistent with morphological transformations reported in previous studies of electrically stimulated nucleated erythrocytes. The spherical cell shape observed in the present study corresponds to the progressive cell rounding reported by Becker and Murray (1967), Smith et al. (1978), and Imani et al. (2019), while the reduced relative cytoplasmic area and increased nuclear prominence correspond to the nuclear enlargement and reduced cytoplasmic area reported by Smith et al. (1978), Chiabrera et al. (1979), and Imani et al. (2019). The progressive loss of cytoplasmic colour and haemoglobin reported by Smith et al. (1978) and Imani et al. (2019) also suggests a reduction in specialised erythrocyte characteristics. Importantly, additional analyses indicated that these morphological changes were accompanied by electrically stimulated RNA and protein synthesis (Harrington and Becker, 1973), RNA-associated staining changes and the apparent involvement of transmembrane calcium movement (Smith et al., 1978), and increased nuclear area and chromatin accessibility without evidence of an initial increase in DNA content (Chiabrera et al., 1979).

Alternative explanations for these changes could include cell death or nonspecific electrical injury. However, the observed changes differ from typical apoptotic and necrotic morphology, while trypan-blue exclusion reported by Imani et al. (2019) argues against immediate loss of viability. These findings support the interpretation that the changes observed in this study represent an active, dedifferentiation-like morphological transformation in the cells.

The present study retained the principal features of Becker and Murray’s experimental framework: amphibian nucleated erythrocytes were diluted with balanced Ringer’s solution, exposed to small direct currents within a non-uniform field chamber and observed continuously by microscopy. However, several methodological differences should be considered. Becker and Murray used fresh heart blood collected from sacrificed frogs and delivered the current using a dry-cell battery, fixed and variable resistors and a microammeter. In the present study, blood was collected from the ventral abdominal vein of living toads, stimulation was delivered using a custom-built precision adjustable current source, and EDTA-treated, lithium-heparin-treated and untreated samples were compared. The present study also defined sample-storage conditions and examined a broader current range.

Overall, the present study reproduced morphological changes similar to those reported by Becker and Murray that may be indicative of dedifferentiation, while also identifying several experimental factors that should be considered when interpreting the findings. Firstly, the changes in the ion concentrations of the sample caused by anti-coagulants must be considered. The potential for chemically inducing cell dedifferentiation is suggested by the dedifferentiation of specific cells resulting from elevated levels of EDTA. It is also important to note that the specific effects of EDTA on voltage-gated channels and induced electrical currents appear to depend on various factors, including the concentration of EDTA, the duration of exposure, and the specific cell types involved. Further research is needed to explore the mechanisms and consequences of EDTA’s action. We theorise that EDTA can affect blood ion levels, potentially impacting ion-controlled gates and induced electrical currents. Calcium ions, in particular, are involved in the opening and closing of ion channels, and their depletion due to the addition of EDTA can interfere with the normal functioning of these gates. Supporting this interpretation, Smith et al. (1978) induced dedifferentiation-like changes using a calcium ionophore and found that these effects could be blocked by ${\text{LaCl}}_{3}$ . This provided evidence that transmembrane ${\text{Ca}}^{2+}$ exchange plays a critical role in the observed transformation. Secondly, any changes between the stimulation chambers used in the experiments will affect the current and its conduction throughout the sample. The exact composition of Ringers’ solution used in the experiments may also influence the speed of the dedifferentiation process. The dedifferentiation process is likely also influenced by cellular age, since cell samples from different frog subjects exhibited slightly different reaction times.

Although ion channels drive vital physiological and pathophysiological processes, their specific role in erythrocytes only recently gained scientific recognition. The mechanisms dictating their signalling, interactions, and coordinated regulation remain largely unexplored, especially regarding the generation of “pseudo action potentials” in red blood cells. However, ion channels have been shown to play a critical role in RBC terminal differentiation, enucleation, and circulation by enabling the cells to pass through capillaries smaller than their diameters. Further research is needed to fully understand the role of ion channels in red blood cell function and their contribution to health and disease (Lindern et al., 2022).

The sequence of morphological changes observed indicates that alterations in the environment lead to modifications in the membrane and underlying cytoplasm. These modifications may transmit information to the nucleus, which subsequently triggers the dedifferentiation of a mature cell, which results in the transformation of the cell into a relatively undifferentiated state. The degree to which these significant changes in cell morphology and function are attributed to genetic derepression mechanisms is still a matter of speculation. The significance of the nonuniformity of the electrical field pattern lies in its role in facilitating the occurrence of dielectrophoresis when dielectric particles are present in such a field. The erythrocyte exhibits a net electrical charge on its surface, suggesting the presence of specific electrically charged groups within its cell membrane. The presence of a nonuniform direct current (DC) field has the potential to cause distortions in the observed pattern. Future research in this field should include investigations of the effects of alternating current (AC) in contrast to DC stimulation. The optimal amplitude and frequency parameters, leading to the most significant results may lead to an optimised control-system for effective generation of pluripotent stem-cells and regeneration of cell types. Additionally, the possibility of redifferentiating dedifferentiated pluripotent stem cells may present an intriguing future study. Dedifferentiated cells could potentially be further transformed into new specialised cells for transplants or regenerative uses. One study by Ramesh et al. (2009) discusses the potential of dedifferentiated/reprogrammed pluripotent stem cells for therapeutic purposes. The authors highlight the need for further studies to refine the technical aspects of dedifferentiation induction and redifferentiation into specific cell types. Future research should explore the use of weak electric fields as a means to guide the redifferentiation process. Multiple independent groups have shown that both mouse and human induced established pluripotent stem (iPS) cells typically maintain the potential to differentiate into derivatives of all germ layers and are epigenetically and transcriptionally similar to normal embryonic stem cells (Amabile and Meissner, 2009).

This proof-of-concept study successfully replicated Becker ad Murray’s foundational findings, confirming that small bioelectrical signals can independently trigger morphological changes suggestive of dedifferentiation in amphibian nucleated erythrocytes. Furthermore, by identifying the influence of environmental variables, such as the use of EDTA and its impact on ion channel dynamics, we clarified critical experimental parameters that affect cellular response times. These results validate a low-barrier model for exploring bioelectrically-induced cellular reprogramming, providing a vital stepping stone for future advancements in regenerative medicine and pluripotent stem cell generation.

## Data Availability

The original contributions presented in the study are included in the article/Supplementary Material, further inquiries can be directed to the corresponding author.
